# Error evaluation of partial scattering functions obtained from contrast-variation small-angle neutron scattering

**DOI:** 10.1107/S1600576724010872

**Published:** 2025-02-01

**Authors:** Koichi Mayumi, Tatsuro Oda, Shinya Miyajima, Ippei Obayashi, Kazuaki Tanaka

**Affiliations:** a Institute for Solid State Physics, University of Tokyo, 5-1-5 Kashiwanoha, Kashiwa-Shi, Chiba, 277-8581, Japan; b Faculty of Science and Engineering, Iwate University, Japan; c Center for Artificial Intelligence and Mathematical Data Science Okayama University,Japan; d Global Center for Science and Engineering Waseda University,Japan; Oak Ridge National Laboratory, USA; North Carolina State University, USA

**Keywords:** small-angle neutron scattering, contrast variation, error evaluation

## Abstract

Contrast-variation small-angle neutron scattering (CV-SANS) is a powerful tool to evaluate the structure of multi-component systems by decomposing the scattering intensities *I* measured with different scattering contrasts into partial scattering functions *S* of self- and cross-correlations between components. The measured *I* contains a measurement error Δ*I*, and Δ*I* results in an uncertainty in the partial scattering functions Δ*S*. However, the error propagation from Δ*I* to Δ*S* has not been quantitatively clarified. In this work, we have established deterministic and statistical approaches to determine Δ*S* from Δ*I*. We have applied the two methods to (i) computational data for a core–shell sphere, and experimental CV-SANS data of (ii) clay/polyethylene glycol aqueous solutions and (iii) polyrotaxane solutions, and have successfully estimated the errors in *S*. The quantitative error estimation in *S* offers a strategy to optimize the combination of scattering contrasts to minimize error propagation.

## Abstract 2

We have established mathematical error estimation methods for structural analysis of multi-component systems by contrast-variation small-angle neutron scattering. Our methods can be used to optimize the selection of scattering contrasts to minimize error propagation.

## 1. Introduction

Contrast-variation small-angle neutron scattering (CV-SANS) has been utilized to study the nano­structure of multicomponent systems, such as organic/inorganic composite materials (Endo *et al.*, 2008 ▸; Takenaka *et al.*, 2009 ▸; Endo *et al.*, 2004 ▸), self-assembled systems of amphipathic molecules (Richter *et al.*, 1997 ▸; Fanova *et al.*, 2024 ▸), complexes of biomolecules (Jeffries *et al.*, 2016 ▸; Nickels *et al.*, 2017 ▸) and supramolecular systems (Mayumi *et al.*, 2009 ▸; Endo *et al.*, 2011 ▸). For isotropic materials, 2D SANS data are converted to a 1D scattering function *I*(*Q*), where *Q* is the magnitude of the scattering vector [*Q* = (4π/λ)sin(θ/2), with θ the scattering angle and λ the wavelength of the incident neutron beam]. In the case of a system with *p* components, the scattering function is the sum of partial scattering functions *S*_*ij*_(*Q*) (Endo, 2006 ▸),
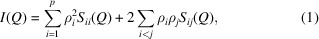
where ρ_*i*_ is the scattering length density of the *i*th component, *S*_*ii*_(*Q*) is a self-term corresponding to the structure of the *i*th component, and *S*_*ij*_(*Q*) is a cross-term originating from the correlation between the *i*th component and *j*th component. On the assumption of incompressibility, equation (1) can be reduced to (Endo, 2006 ▸)
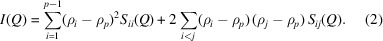
For three-component systems in which two solutes (*i* = 1, 2) are dissolved in a solvent (*i* = 3), the scattering function is given as
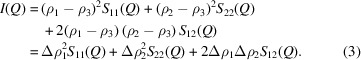
Here, Δρ_*i*_ is the scattering length density difference between the *i*th solute and the solvent. On the basis of equation (3), by measuring *I*(*Q*) of *N* samples (*N* ≥ 3) with different scattering contrasts (Δρ_1_ and Δρ_2_), it is possible to determine the three partial scattering functions *S*_11_(*Q*), *S*_22_(*Q*) and *S*_12_(*Q*):
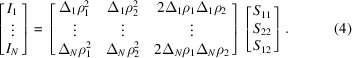
From the calculated partial scattering functions, we can analyze the structure of each solute and the cross-correlation between the two solutes. Despite the usefulness of CV-SANS, its application has been limited due to the complexity and uncertainty of this calculation. The experimentally obtained *I*(*Q*) has a statistical error Δ*I* and therefore we should consider how Δ*I* propagates to the error in *S*(*Q*):
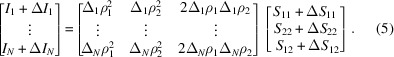
However, as far as we know, the relationship between Δ*I* and Δ*S* has not been clarified. The contribution of the present study is an estimation of the transition from Δ*I* to Δ*S*. To achieve this, we adopted two approaches: deterministic and statistical error estimation.

The objective of deterministic error estimation is to estimate analytically the upper bounds of |Δ*S*_11_|, |Δ*S*_12_| and |Δ*S*_22_| in equation (5). This essentially amounts to quantitative clarification of the sensitivity of *S* to the variations in *I*. It is essential to recognize this relationship because it has a direct impact on the precision required for observing *I*. Theoretically, solving a least-squares problem is equivalent to multiplying its right-hand-side vector by the Moore–Penrose inverse [see *e.g.* Golub & Van Loan (2013 ▸)], which is a kind of generalized inverse of its coefficient matrix. Therefore, the Moore–Penrose inverse plays a vital role in this analysis, enabling a detailed examination of the impact of each input variable. This sheds light on the complex pathways of error propagation within a system. Such an error estimation has already been done in the context of verified numerical computation [see *e.g.* Miyajima (2014 ▸)] and its effectiveness has been confirmed.

The objective of statistical error estimation is to establish the probabilistic description of Δ*S* from data under some statistical assumptions. Statisticians have long considered the problem of error estimation, such as interval estimation (Dekking *et al.*, 2005 ▸) and Bayesian statistics (Hoff, 2009 ▸). Recently, this field has been referred to as uncertainty quantification (Sullivan, 2015 ▸) and has been widely studied. The present study applies basic Bayesian inference with a non-informative prior distribution to estimate Δ*S*. The estimation assumes that the error in *I*, namely Δ*I*, follows a normal distribution. The framework is also used to examine the robustness of the estimation.

These approaches effectively capture the inherent uncertainties in the observational errors. The error bounds are accurately derived from the mathematical structure of equation (4), thereby offering significant insights into the reliability and precision of the computational results for the partial scattering functions. In this study, we applied our error estimation methods to (i) computational data of a core–shell sphere and experimental CV-SANS data of (ii) clay/polyethylene glycol (PEG) aqueous solutions and (iii) polyrotaxane solutions (Mayumi *et al.*, 2009 ▸). Polyrotaxane is a topological supramolecular assembly, in which ring molecules are threaded onto a linear polymer chain. This study demonstrates the effectiveness of our method in quantifying uncertainties arising from the randomness of observational errors. In both error estimation approaches, the condition number of the coefficient matrix is a useful tool to evaluate the degree of error propagation (see Sections 2.1 and 2.2 for further detail).

## 2. Methods

### 2.1. Deterministic error estimation

As mentioned above, despite the usefulness of CV-SANS, its application has been limited due to the statistical error Δ*I* associated with the experimentally obtained *I*(*Q*). How Δ*I* propagates to the error in *S*(*Q*) has not been well studied. To address this issue, this subsection presents a theory that clarifies how Δ*I* propagates to the error in *S*(*Q*), Δ*S*, in a deterministic sense. The theory in this subsection is based on error analyses in numerical computations (Higham, 2002 ▸).

We define 
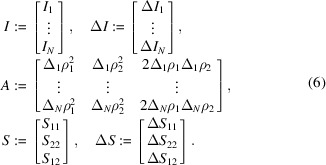
Then, (5) can be written as 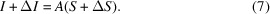
It is obvious that *S* and Δ*S* are 3D vectors and *A* is an *N* × 3 matrix. Sections 2.1 and 2.2 generalize equation (5) and treat the case where *S* and Δ*S* are *m*-dimensional vectors and *A* is an *N* × *m* matrix. To this end, we introduce here the notation used in Sections 2.1 and 2.2. For 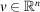, let *v*_*i*_ for *i* = 1,…, *n* be the *i*th component of *v* and 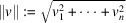. For 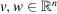, the inequality *v* ≤ *w* means that *v*_*i*_ ≤ *w*_*i*_ holds for all *i*. Let 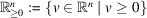. For 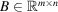, let *b*_*ij*_ and *∥B∥* be the (*i*, *j*) element and 2-norm [see *e.g.* Golub & Van Loan (2013 ▸)] of *B*, respectively. We suppose *v* ≤ *w* and define 
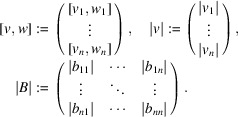
We denote the Moore–Penrose inverse of *B* by *B*^+^. When *m* ≥ *n* and *B* has full column rank in particular, we have *B*^+^ = (*B*^T^*B*)^−1^*B*^T^, where *B*^T^ denotes the transpose of *B*.

We present Theorem 1[Statement theorem1] for clarifying how Δ*I* propagates to Δ*S* in a deterministic sense. See Appendix *A*[App appa] for its proof. Theorem 1[Statement theorem1] says that we can analytically estimate an upper bound on |Δ*S*|.

Theorem 1 Let 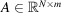, 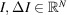, 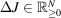, 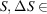

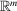
 and Δ*T*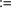 |*A*^+^|Δ*J*. Suppose that *N* ≥ *m*, *AS* = *I*, *I* + Δ*I* = *A*(*S* + Δ*S*), |Δ*I*| ≤ Δ*J* and *A* has full column rank. It then follows that 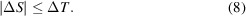

Remark 1 In practice, we regard the standard deviation of the experimentally obtained *I*(*Q*) as Δ*J*.

We define the condition number cond(*A*) by cond(*A*) 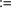 ∥*A*∥∥*A*^+^∥. Let 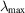 and 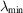 be the largest and smallest singular values of *A*, respectively. We then have 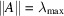 and 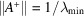, so that 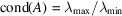. Using the singular value decomposition [see *e.g.* Golub & Van Loan (2013 ▸)] of *A*^+^, it can be shown that 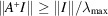. These relations together with 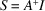 and 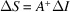 give 
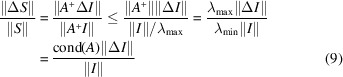
if *S* ≠ 0 and *I* ≠ 0. This inequality implies that ∥Δ*S*∥/∥*S*∥ is enlarged by cond(*A*), suggesting that cond(*A*) is an important parameter related to the degree of error propagation from *I* to *S*. A more detailed explanation of cond(*A*) is given in Appendix *B*[App appb].

### 2.2. Statistical error estimation

To quantify the estimation errors Δ*S*_*ij*_ statistically, we rewrite (5) into the following statistical model: 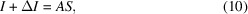
where 
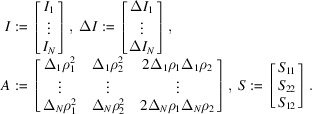
In this formulation, *S*_*ij*_ includes Δ*S*_*ij*_ since the values we want to estimate are considered random variables with a prior distribution in the Bayesian framework. The following assumptions are made to build the statistical model.

(i) Each Δ*I*_*i*_ is a normal random variable with mean zero and standard deviation σ_*i*_.

(ii) Δ*I*_1_,…, Δ*I*_*N*_ are probabilistically independent.

(iii) The prior distribution of *S* is a multivariate normal distribution 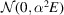, where α > 0 is a parameter and *E* is an *N* × *N* identity matrix.

From the first two assumptions, Δ*I* is a multivariate normal random variable with mean zero and covariance matrix Σ, where 
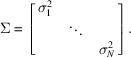
Then the posterior distribution of *S* is 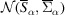 from Bayes’ formula for multivariate normal distributions [Section 6.1 of Sullivan (2015 ▸)], where 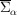 = 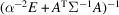 and 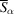 = 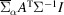. The prior distribution represents the assumption on the scale of *S* and we try to remove the effect of the assumption using a non-informative prior by having α → ∞. As a result, the posterior distribution of *S* is 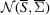, where 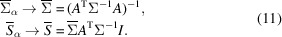
By setting 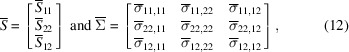
the result can be interpreted as follows:

(i) The posterior distribution of *S*_11_ is a normal distribution with mean 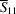 and variance 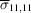. This means that 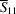 is the most likely value of *S*_11_, but the uncertainty of the estimation is described by the normal distribution whose variance is 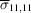.

(ii) *S*_22_ and *S*_12_ can also be evaluated in the same way.

(iii) A non-diagonal element of 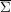 is the covariance of estimated values; that is, the estimated values are correlated.

We remark that 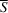 differs from the solution of the standard least-squares problem, *A*^+^*I*. In fact, 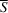 is the solution of the weighted least-squares problem: argmin_*S*_∥Σ^−1^(*I* − *AS*)∥. This formula means that σ_*j*_ quantifies the reliability of measurements and squared errors are weighted by the reliability factors.

The following theorem is useful for estimating the error before an experiment, since singular values can be calculated only from *A*. See Appendix *A*[App appa] for its proof.

Theorem 2

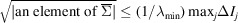
, where 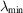 is the smallest singular value of the matrix *A*.

Because the diagonal elements of 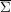 are the standard deviations of *S*_11_, *S*_22_ and *S*_12_, we can say that the absolute error is roughly scaled by 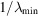. Because the observation *I* is roughly equal to 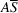, we can estimate the relation between ∥*I*∥ and 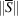 as follows: 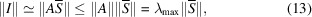
where 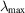 is the largest singular value of the matrix *A*. This means that 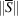 is approximately bounded by 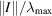 from below and we can say that the relative error is roughly scaled by 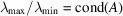. This suggests that condition numbers are useful for estimating the relative errors from the viewpoint of Bayesian statistics.

Using this formulation, we can examine the robustness of the estimation. The assumptions of model (10) are not perfect and a real measurement has unknown error factors, such as uncertainty in the scattering length density, deviation of the noise distribution from the normal distribution and unknown bias of the measurement device. Of course, such errors are expected to be very small, but if these small errors significantly disturb the result, the estimated result will not be reliable.

One of the simplest ways to check the robustness of the result is to extend the error bars of the measurement virtually. Here, we consider what happens when σ_1_,…, σ_*N*_ are multiplied by μ > 1. In this case, Σ is multiplied by μ^2^ in (11), and as a result 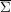 is multiplied by μ^2^ but 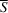 is not changed. Therefore, the standard deviations of the posterior distributions are multiplied by μ, which means that the estimation’s uncertainty is enlarged by μ.

### 2.3. Computational data for a core–shell sphere

**Figure 1:**
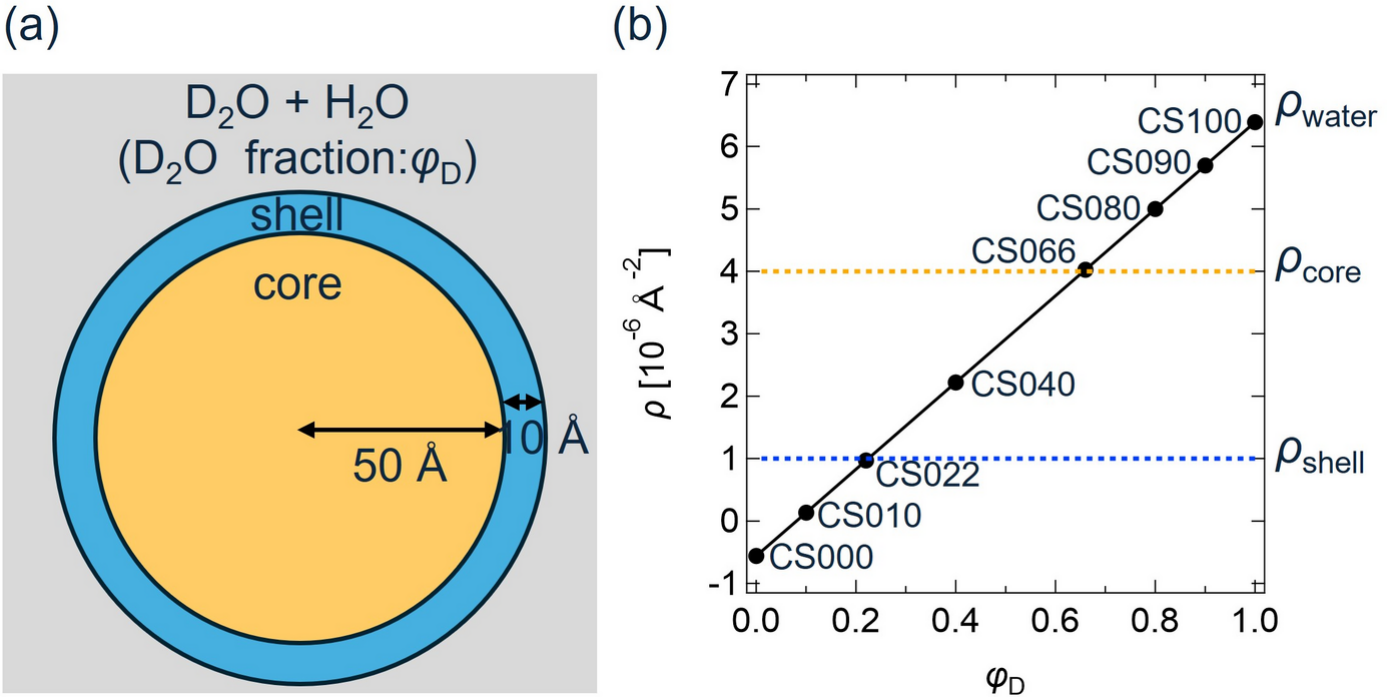
(*a*) Schematic illustration of a core–shell sphere dispersed in a D_2_O/H_2_O mixture. (*b*) Scattering length densities of the core (ρ_core_), shell (ρ_shell_) and solvent (ρ_water_) plotted against the D_2_O fraction of the solvent φ_D_.

To verify the validity of the deterministic and statistical error estimations, we first applied the two methods to computational data for a core–shell sphere [Fig. 1 ▸(*a*)]. We computed the scattering intensities *I*(*Q*) for core–shell spheres dispersed in D_2_O/H_2_O mixtures with different D_2_O fractions using the ‘Core shell sphere’ model of the *SASview* software (Version 5.0.6; https://www.sasview.org/). The core radius and shell thickness were 50 and 10 Å, respectively. While the scattering length densities of the core and shell were fixed at 4.0 × 10^−6^ and 1.0 × 10^−6^ Å^−2^, respectively, the scattering length density of the solvent was changed with differing D_2_O fraction φ_D_ as follows (Endo *et al.*, 2008 ▸): 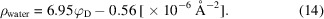
The core–shell samples with φ_D_ = 1.0, 0.90, 0.80, 0.66, 0.40, 0.22, 0.10 and 0.0 are named as CS100, CS090, CS080, CS066, CS040, CS022, CS010 and CS000, respectively [Fig. 1 ▸(*b*)].

### 2.4. CV-SANS data for clay/PEG aqueous solutions and polyrotaxane solutions

**Figure 2:**
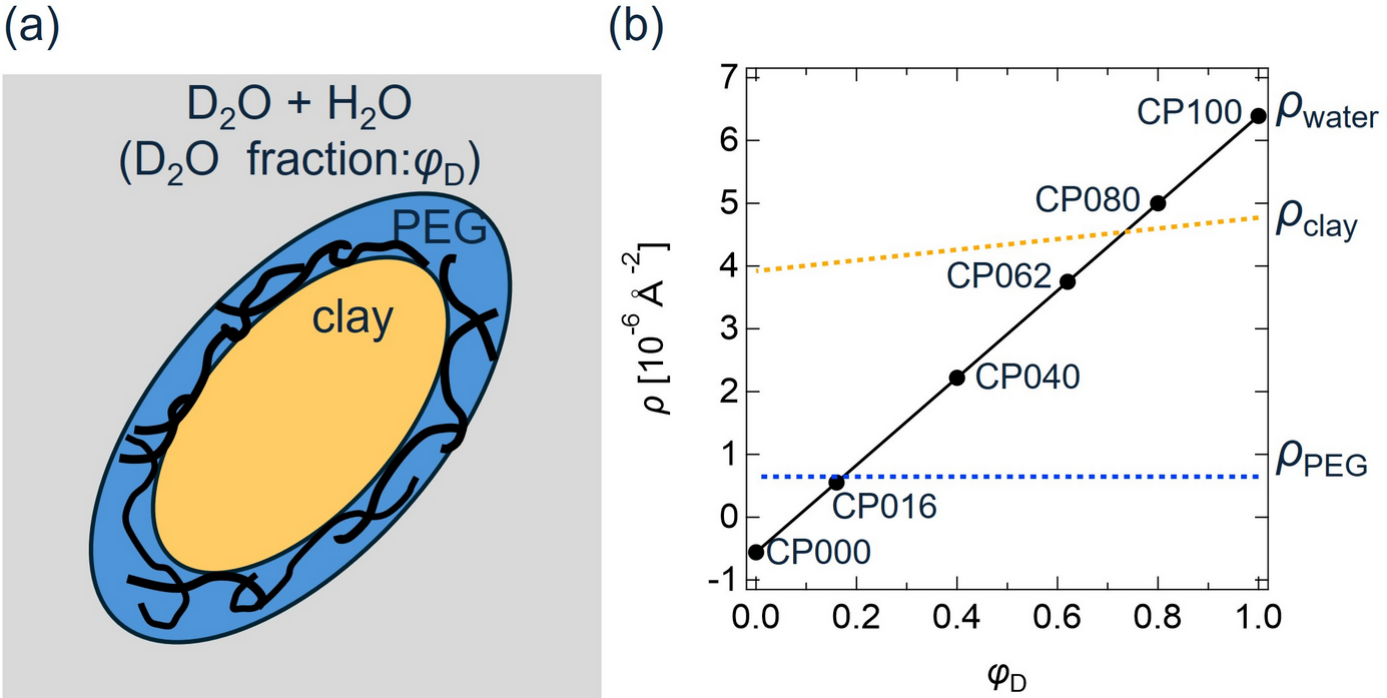
(*a*) Schematic illustration of a clay/PEG solution dissolved in a D_2_O/H_2_O mixture. (*b*) Scattering length densities of the clay (ρ_clay_), PEG (ρ_PEG_) and solvent (ρ_water_) plotted against the D_2_O fraction of the solvent φ_D_.

**Figure 3:**
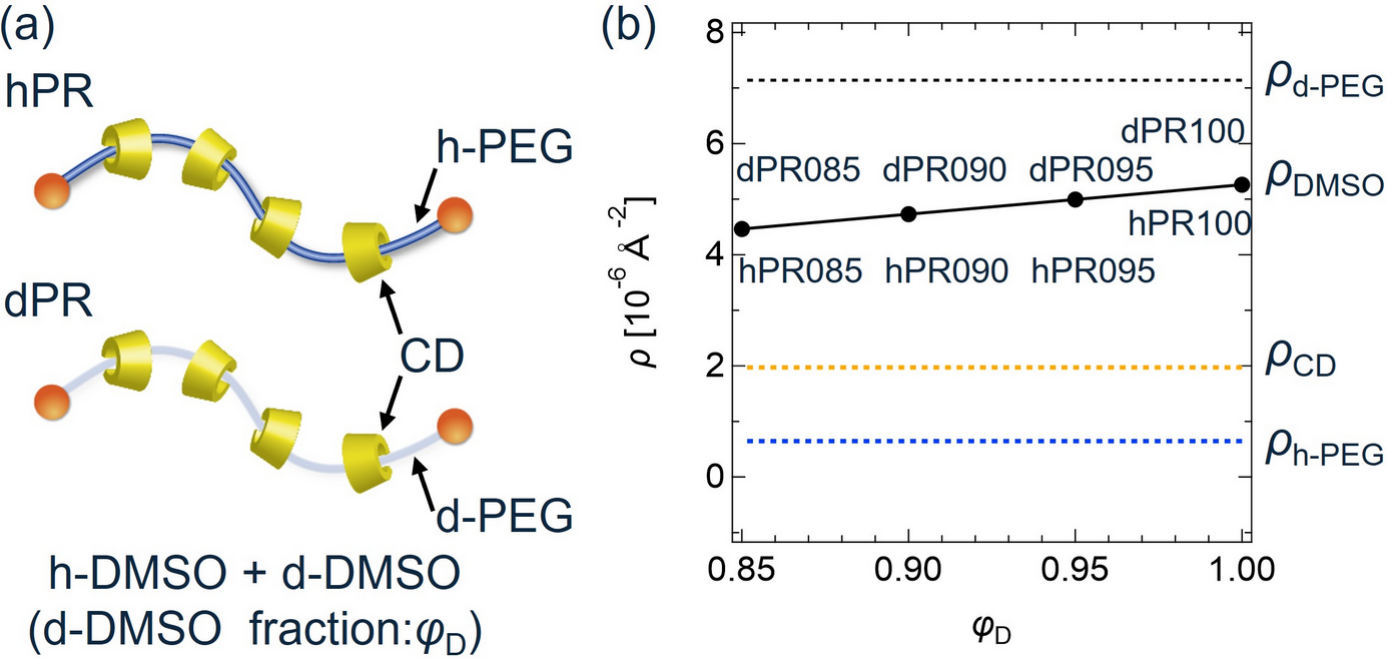
(*a*) Schematic illustration of a PR solution dissolved in a d-DMSO/h-DMSO mixture. (*b*) Scattering length densities of h-PEG (ρ_h-PEG_), d-PEG (ρ_d-PEG_), CD (ρ_CD_) and solvent (ρ_DMSO_) plotted against the d-DMSO fraction of the solvent φ_D_.

For the next step, the error estimation methods were applied to two sets of experimental CV-SANS data: (i) clay/PEG solutions [Fig. 2 ▸(*a*)] and (ii) polyrotaxane (PR) solutions [Fig. 3 ▸(*a*)]. The clay/PEG solutions were prepared by dissolving Laponite XLG nanoclay (BYK) and PEG (*M*_w_ = 35000, Fluka) in D_2_O/H_2_O mixtures with different D_2_O fractions. According to a previous CV-SANS study of clay/PEG aqueous solutions (Matsunaga *et al.*, 2010 ▸), PEG is adsorbed onto the surface of the clay particles and a core–shell structure is formed as shown in Fig. 2 ▸(*a*). The volume fractions of clay and PEG were 2% and 2.5%, respectively. The volume fraction of D_2_O in the solvent, φ_D_, was changed to vary the scattering contrasts of the clay and PEG. Corresponding to φ_D_ = 1.0, 0.80, 0.62, 0.40, 0.16 and 0.00, the clay/PEG solutions are named as CP100, CP080, CP062, CP040, CP016 and CP000, respectively [Fig. 2 ▸(*b*)]. The scattering length density of PEG, ρ_PEG_, is 0.65 × 10^−6^ Å^−2^, while the scattering length densities of the D_2_O/H_2_O mixtures vary with φ_D_ as described in equation (14). The scattering length density of the clay particle, ρ_clay_, is given as follows (Endo *et al.*, 2008 ▸): 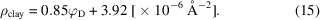


The CV-SANS data for the PR solutions are reported in our previous paper (Mayumi *et al.*, 2009 ▸). For the CV-SANS measurements of PR solutions, we used PR consisting of hydrogenated (h-) PEG or deuterated (d-) PEG as a linear polymer chain and α-cyclodextrins (CDs) as rings [Fig. 3 ▸(*a*)]. The scattering length densities ρ of h-PEG, d-PEG and CD were 0.65 × 10^6^, 7.1 × 10^6^ and 2.0 × 10^6^ Å^−2^, respectively. h-PR and d-PR were dissolved in mixtures of hydrogenated dimethyl sulfoxide (h-DMSO) and deuterated DMSO (d-DMSO). The volume fraction of PR in the solutions was 8%. The volume fractions of d-DMSO in the solvent φ_D_ were 1.0, 0.95, 0.90 and 0.85, and the corresponding scattering length densities of the solvents were 5.3 × 10^6^, 5.0 × 10^6^, 4.7 × 10^6^ and 4.5 × 10^6^ Å^−2^, respectively. Depending on the d-DMSO fraction and type of PR, the PR solutions are named as hPR100, hPR095, hPR090, hPR085, dPR100, dPR095, dPR090, dPR085, as shown in Fig. 3 ▸(*b*).

The SANS measurements of the clay/PEG and PR solutions were performed at 298 K using the SANS-U diffractometer of the Institute for Solid State Physics, University of Tokyo, located at the JRR-3 research reactor of the Japan Atomic Energy Agency in Tokai, Japan. The incident beam wavelength was 7.0 Å and the wavelength distribution was 10%. The sample-to-detector distances were 1 and 8 m for the clay/PEG solutions and 1 and 4 m for the PR solutions. The scattered neutrons were collected with a 2D detector and then the necessary corrections were made, such as air and cell scattering subtractions. After these corrections, the scattered intensity was normalized to the absolute intensity using a standard polyethylene film with known absolute scattering intensity. The 2D intensity data were circularly averaged and the incoherent scattering was subtracted. The averaged scattering intensities *I* were plotted against the magnitude of the scattering vector *Q*. The error bars of *I*(*Q*) were given by Δ*I* = ±σ, where σ represents the standard deviation of the circular averaging.

## 3. Results

### 3.1. Error estimation for computational data of core–shell sphere

**Figure 4:**
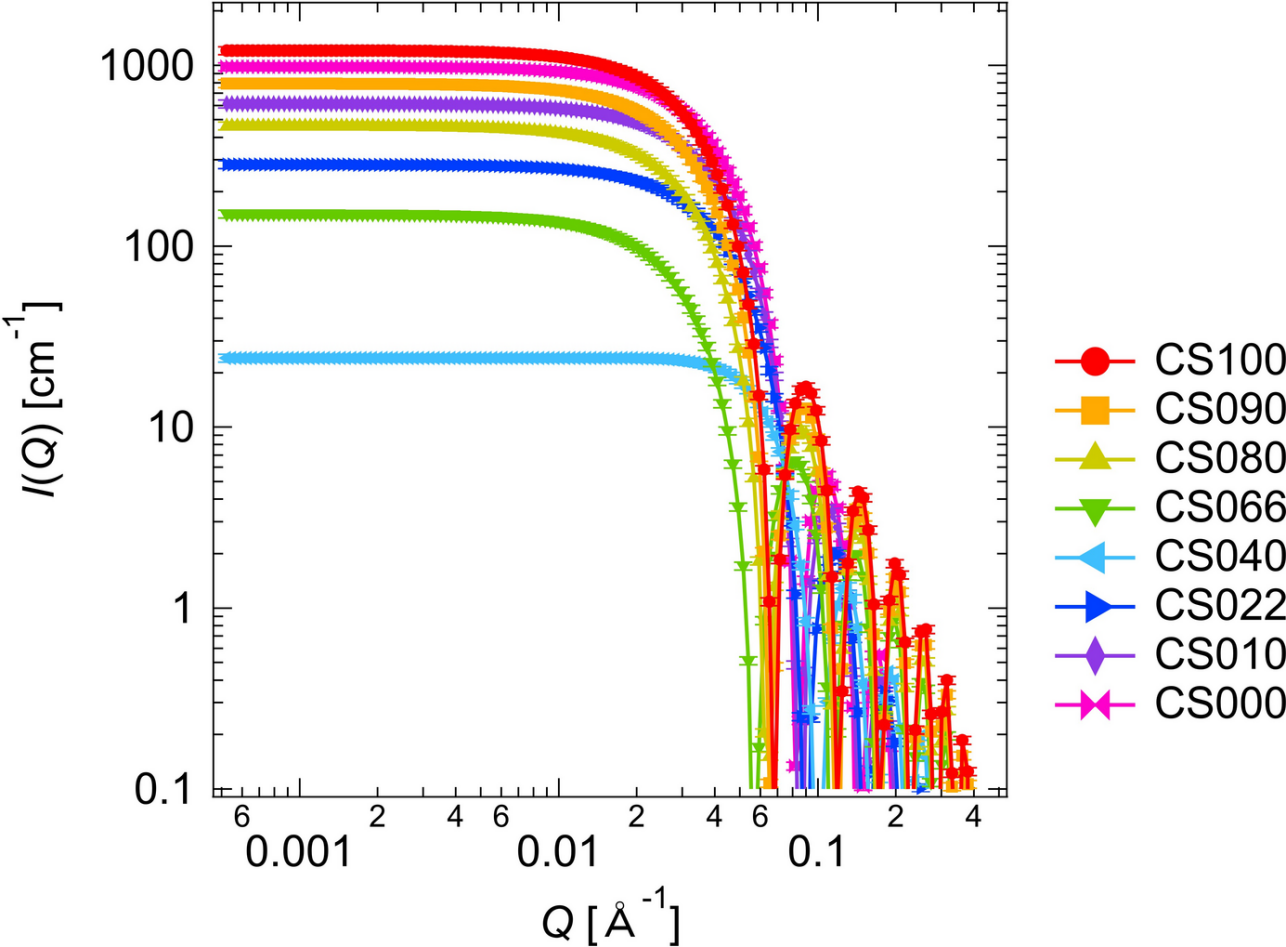
Computed scattering intensities *I*(*Q*), with error bars, for the core–shell sphere: CS100, CS090, CS080, CS066, CS040, CS022, CS010 and CS000. The error bars are given by Δ*I*(*Q*)/*I*(*Q*) = ±0.05

The computed scattering intensities *I*(*Q*) of the core–shell sphere are shown in Fig. 4 ▸. The relative error in *I*(*Q*), Δ*I*(*Q*)/*I*(*Q*), is set at ±0.05, giving the error bars in Fig. 4 ▸.

The scattering intensity *I*(*Q*) of the core–shell sphere is represented as 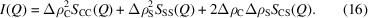
Here, *S*_CC_(*Q*) is the self-term of the core, *S*_SS_(*Q*) is the self-term of the shell and *S*_CS_(*Q*) is the cross-term between the core and shell. This section considers the case when *S*_11_, *S*_22_, *S*_12_, Δ_*i*_ρ_1_ and Δ_*i*_ρ_2_ for *i* = 1,…, *N* in equation (5) correspond to *S*_CC_, *S*_SS_, *S*_CS_, Δρ_C_ and Δρ_S_, respectively.

#### 3.1.1. Deterministic error estimation of core–shell sphere

In this section, we present the results of the deterministic error estimation described in Section 2.1. Let *I*, Δ*I*, *A*, *S* and Δ*S* be as defined in equation (6). Here, the vector Δ*J* in Theorem 1[Statement theorem1] is 0.05*I*.

**Figure 5:**
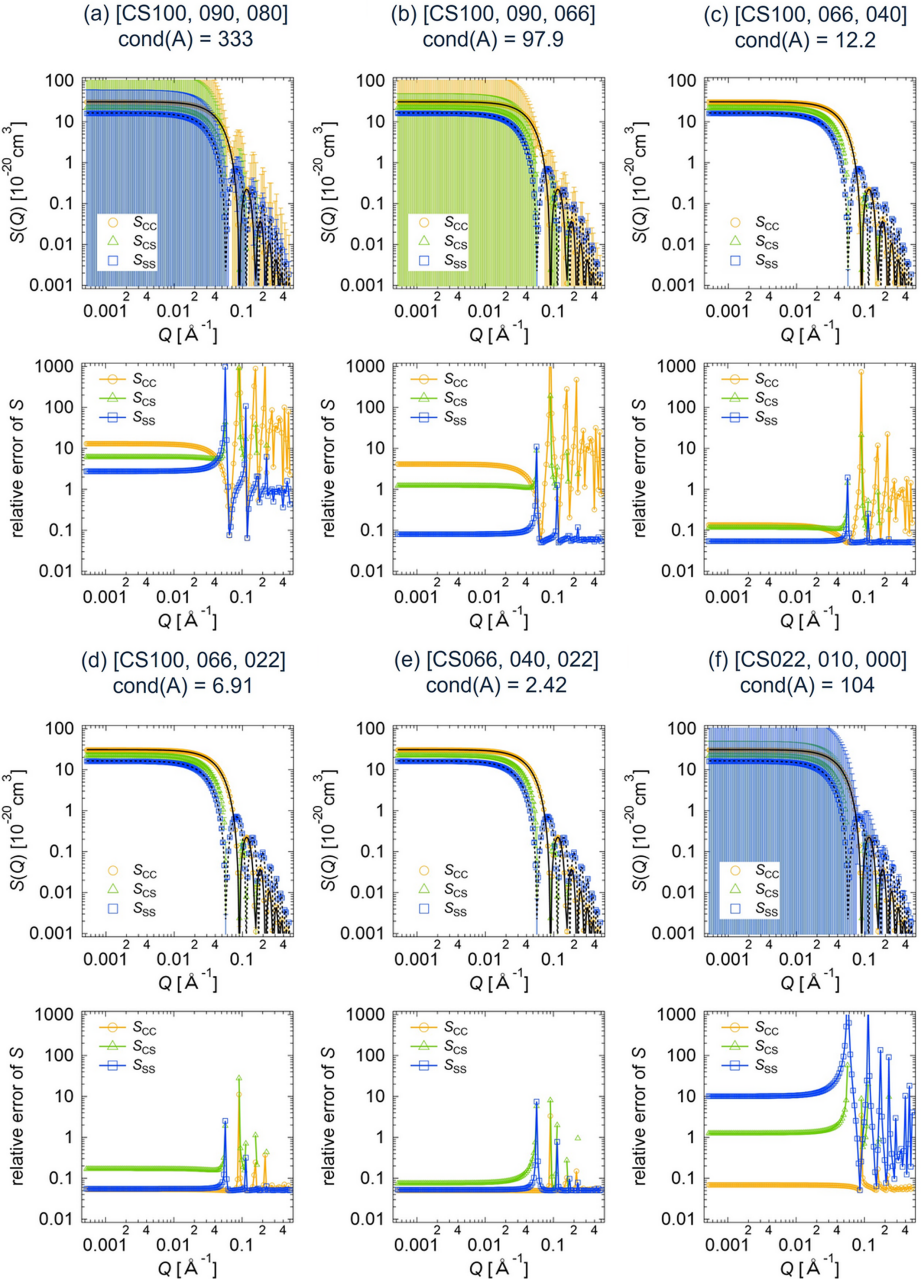
Partial scattering functions with error bars and their relative errors for the core–shell sphere, obtained by applying the deterministic error estimation method to the different data sets of the computed scattering intensities: (*a*) CS100, CS090, CS080; (*b*) CS100, CS090, CS066; (*c*) CS100, CS066, CS040; (*d*) CS100, CS066, CS022; (*e*) CS066, CS040, CS022; (*f*) CS022, CS010, CS000. The term cond(*A*) is the condition number of the matrix *A*. The black solid and dotted lines correspond to 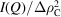 of CS022 (shell matching) and 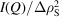 of CS066 (core matching), respectively.

Using various combinations of three scattering intensities out of the eight data sets shown in Fig. 4 ▸, we calculated the partial scattering functions and their errors for the core–shell sphere. We denote the upper bounds on |Δ*S*_CC_|, |Δ*S*_SS_| and |Δ*S*_CS_| obtained according to Theorem 1[Statement theorem1] by Δ*T*_CC_, Δ*T*_SS_ and Δ*T*_CS_, respectively. Figs. 5 ▸(*a*)–5 ▸(*f*) display the numerically computed partial scattering functions, *S*_CC_ + Δ*S*_CC_, *S*_SS_ + Δ*S*_SS_ and *S*_CS_ + Δ*S*_CS_. The error bars for the partial scattering functions are given by Δ*T*_CC_, Δ*T*_SS_ and Δ*T*_CS_. Fig. 5 ▸ also shows the relative errors in the partial scattering functions, defined as Δ*T*_CC_/(*S*_CC_ + Δ*S*_CC_), Δ*T*_SS_/(*S*_SS_ + Δ*S*_SS_) and Δ*T*_CS_/(*S*_CS_ + Δ*S*_CS_).

The calculated partial scattering functions are identical regardless of contrast combinations. Additionally, note that *S*_CC_(*Q*) and *S*_SS_(*Q*) completely overlap with 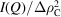 for CS022 (shell matching, black solid lines in Fig. 5 ▸) and 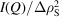 for CS066 (core matching, black dotted lines in Fig. 5 ▸), respectively, indicating the validity of our calculation results. The relative errors in *S* vary with different contrast combinations corresponding to various condition numbers of the matrix *A*, cond(*A*), as shown in Fig. 5 ▸.

#### 3.1.2. Statistical error estimation of core–shell sphere

**Figure 6:**
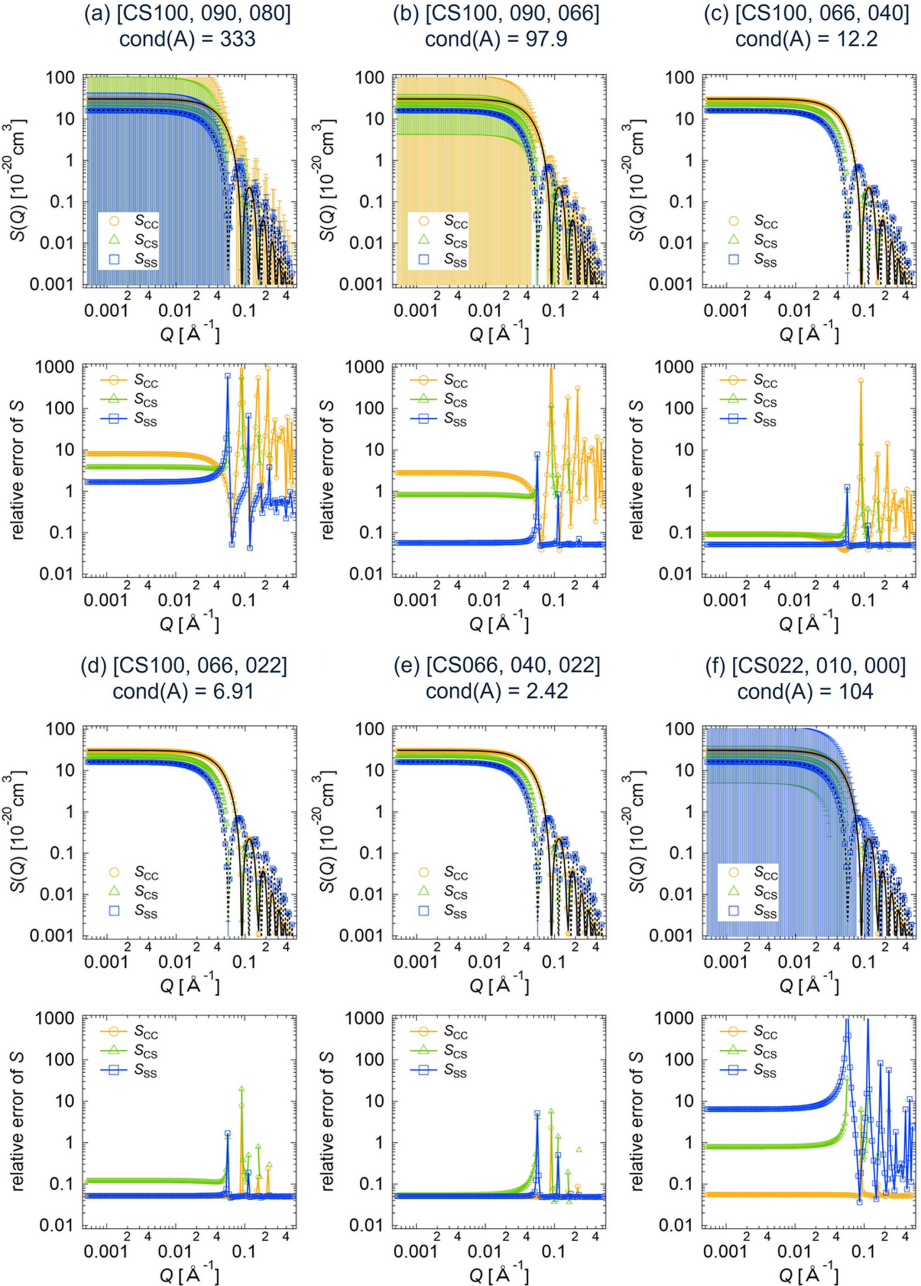
Partial scattering functions with error bars and their relative errors for the core–shell sphere, obtained by applying the statistical error estimation method to the different data sets of the computed scattering intensities: (*a*) CS100, CS090, CS080; (*b*) CS100, CS090, CS066; (*c*) CS100, CS066, CS040; (*d*) CS100, CS066, CS022; (*e*) CS066, CS040, CS022; (*f*) CS022, CS010, CS000. The black solid and dotted lines correspond to 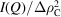 of CS022 (shell matching) and 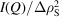 of CS066 (core matching), respectively.

Next, we applied the statistical method described in Section 2.2 to the computational core–shell sphere data by setting *I*_1_,…, *I*_*N*_ to the computed scattering intensities and σ_1_,…, σ_*N*_ to the artificial errors, 0.05*I*. Fig. 6 ▸ shows the estimated partial scattering functions and their errors computed by equation (11) for the different contrast combinations (*a*) to (*f*). The error bars represent 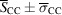, 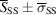 and 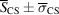, where 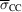 = 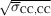, 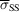 = 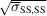 and 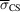 = 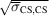. Similarly to the results of the deterministic estimation, the calculated partial scattering functions are the same for all the contrast combinations, and the obtained *S*_CC_(*Q*) and *S*_SS_(*Q*) are identical to, respectively, 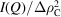 of CS022 (shell matching, black solid lines in Fig. 6 ▸) and 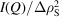 of CS066 (core matching, black dotted lines in Fig. 6 ▸). Fig. 6 ▸ also shows the relative estimated errors in the partial scattering functions, namely 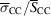, 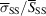 and 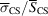.

#### 3.1.3. Comparison between deterministic and statistical error estimation results for core–shell sphere

**Figure 7:**
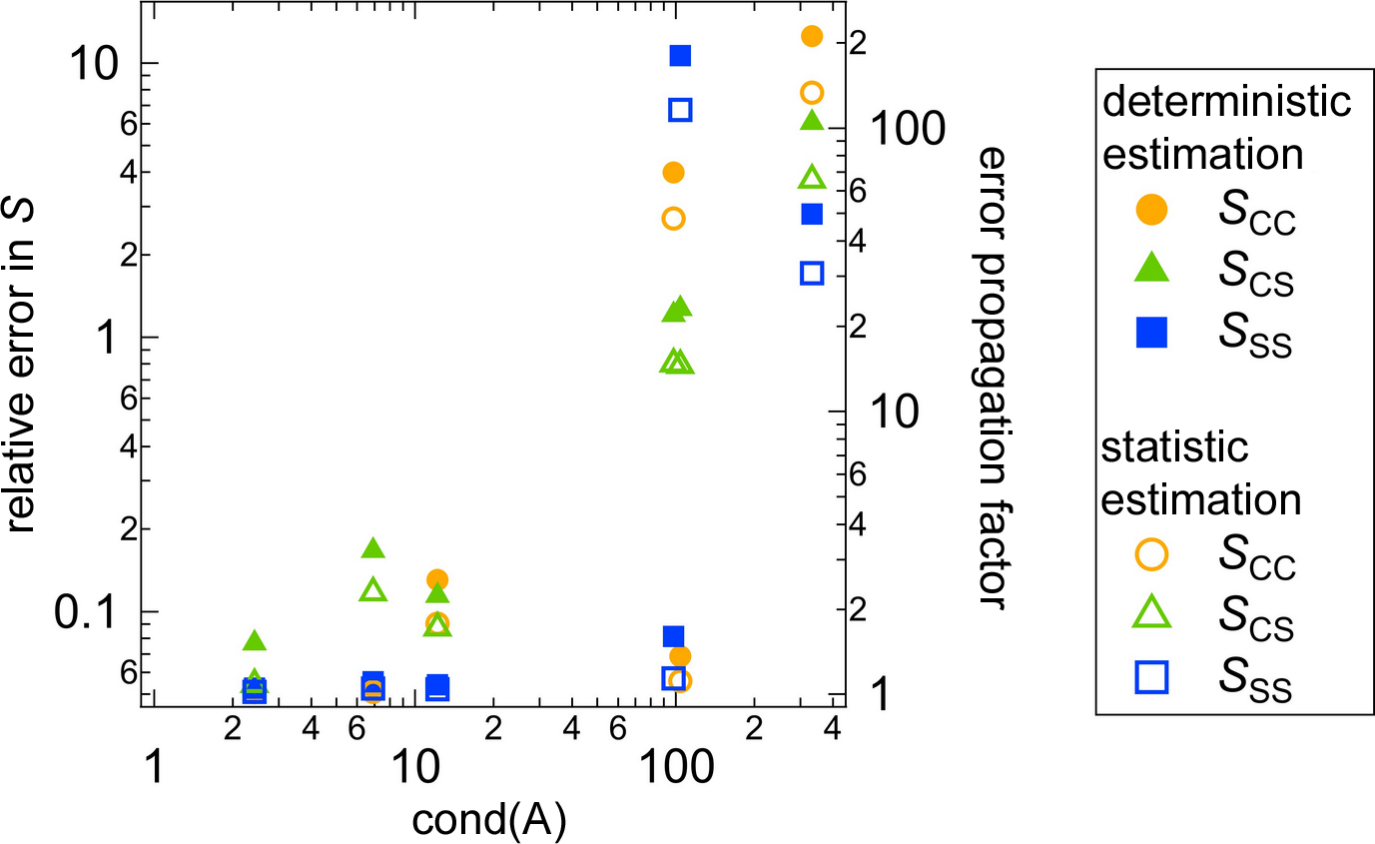
Plot of the relative errors in *S* versus the condition number of *A* for the core–shell sphere system. The right-hand axis represents the error propagation factor.

We compare the results obtained from the determinisic error estimation (Fig. 5 ▸) and the statistical error estimation (Fig. 6 ▸). In Fig. 7 ▸, the relative errors in the partial scattering functions *S* at *Q* = 0.01 Å^−1^ are plotted against the condition number of *A*. The statistical estimation yields smaller relative errors in *S* than the deterministic estimation. This difference in error propagation may result from the different assumptions underlying the two methods. The deterministic error estimation requires weaker assumptions than the statistical method (see Sections 2.1 and 2.2), which results in the larger relative errors in *S* obtained from the deterministic method.

Fig. 7 ▸ shows a positive correlation between the relative error in *S* and the condition number of *A*. This suggests that increasing the condition number of *A* results in increasing the degree of error propagation from the scattering intensities to the partial scattering functions, which is consistent with the explanation of the condition number shown in Appendix *B*[App appb]. We define the error propagation factor as the relative error in *S* divided by the relative error in *I*, 0.05. The right-hand axis of Fig. 7 ▸ represents this error propagation factor. When the condition number of *A* is at its minimum value, 2.42, the error propagation factors for all the partial scattering functions are close to 1, indicating that the error propagation is minimized and the partial scattering functions are accurately determined. However, for the maximum condition number of *A*, 333, the error propagation factors range from 30 to 200, resulting in the large relative errors in *S* exceeding 1 and the large error bars for *S* shown in Figs. 5 ▸(*a*) and 6 ▸(*a*).

### 3.2. Error estimation for experimental data of clay/PEG solutions

**Figure 8:**
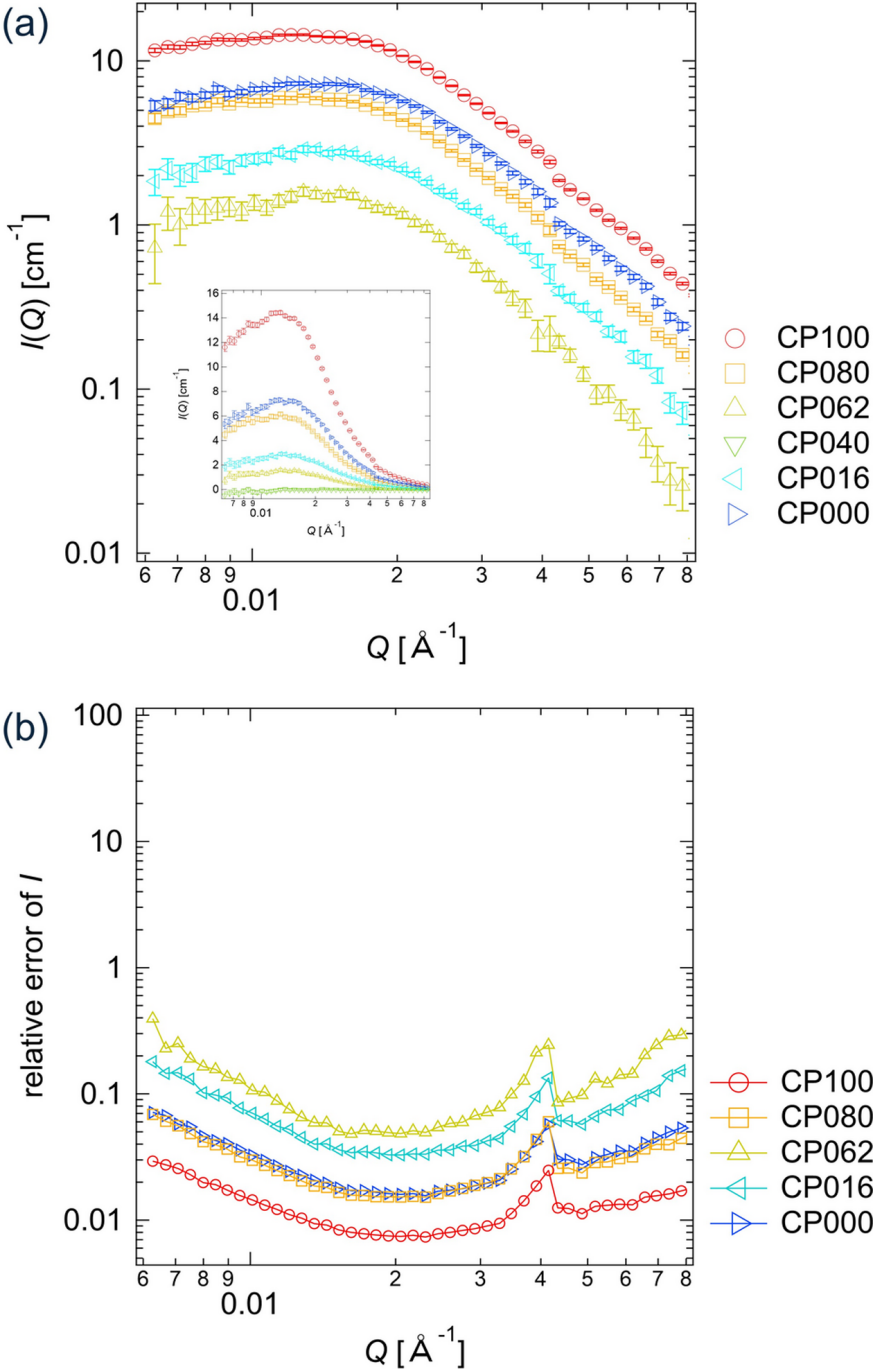
(*a*) Experimentally measured scattering intensities *I*(*Q*) with error bars and (*b*) relative errors in *I*(*Q*) for the clay/PEG solutions: CP100, CP080, CP062, CP040, CP016 and CP000. In the inset of panel (*a*), linear *I*(*Q*) is plotted against log *Q*.

The experimentally measured scattering intensities *I*(*Q*) of the clay/PEG solutions with various D_2_O fractions are shown in Fig. 8 ▸(*a*). As described in Section 2.4, the error bars for *I*(*Q*) are given by Δ*I* = ±σ, in which σ is the standard deviation of the circular averaging. The relative errors in *I*(*Q*), σ/*I*(*Q*), are shown in Fig. 8 ▸(*b*).

The scattering intensities *I*(*Q*) of the clay/PEG solutions are given by
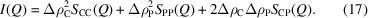
Here, *S*_CC_(*Q*) is the self-term of the clay particles, *S*_PP_(*Q*) is the self-term of PEG and *S*_CP_(*Q*) is the cross-term between the clay and PEG. In this section, *S*_11_, *S*_22_, *S*_12_, Δ_*i*_ρ_1_ and Δ_*i*_ρ_2_ for *i* = 1,…, *N* in equation (5) are represented as *S*_CC_, *S*_PP_, *S*_CP_, Δρ_C_ and Δρ_P_, respectively.

#### 3.2.1. Deterministic error estimation of clay/PEG solutions

We applied the deterministic error estimation method to the CV-SANS experimental data for the clay/PEG solutions. For 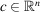 and 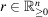, we define 〈*c*, *r*〉 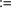 [*c* − *r*, *c* + *r*]. Let *I*, Δ*I*, *A*, *S* and Δ*S* be as defined in equation (6). As mentioned in Remark 1[Statement enun1], we consider the standard deviation of the experimentally obtained *I*(*Q*) as Δ*J* in Theorem 1[Statement theorem1]. Consequently, the interval 〈*I* + Δ*I*, Δ*J*〉 contains *I* with a probability of approximately 68.3% if *I* follows a normal distribution. Because Δ*T* in Theorem 1[Statement theorem1] represents the upper bound on |Δ*S*|, the interval 〈*S* + Δ*S*, Δ*T*〉 contains *S* with a probability equal to or greater than 68.3% in this case. If *I* ∈ 〈*I* + Δ*I*, Δ*J*〉 holds rigorously, then *S* ∈ 〈*S* + Δ*S*, Δ*T*〉.

**Figure 9:**
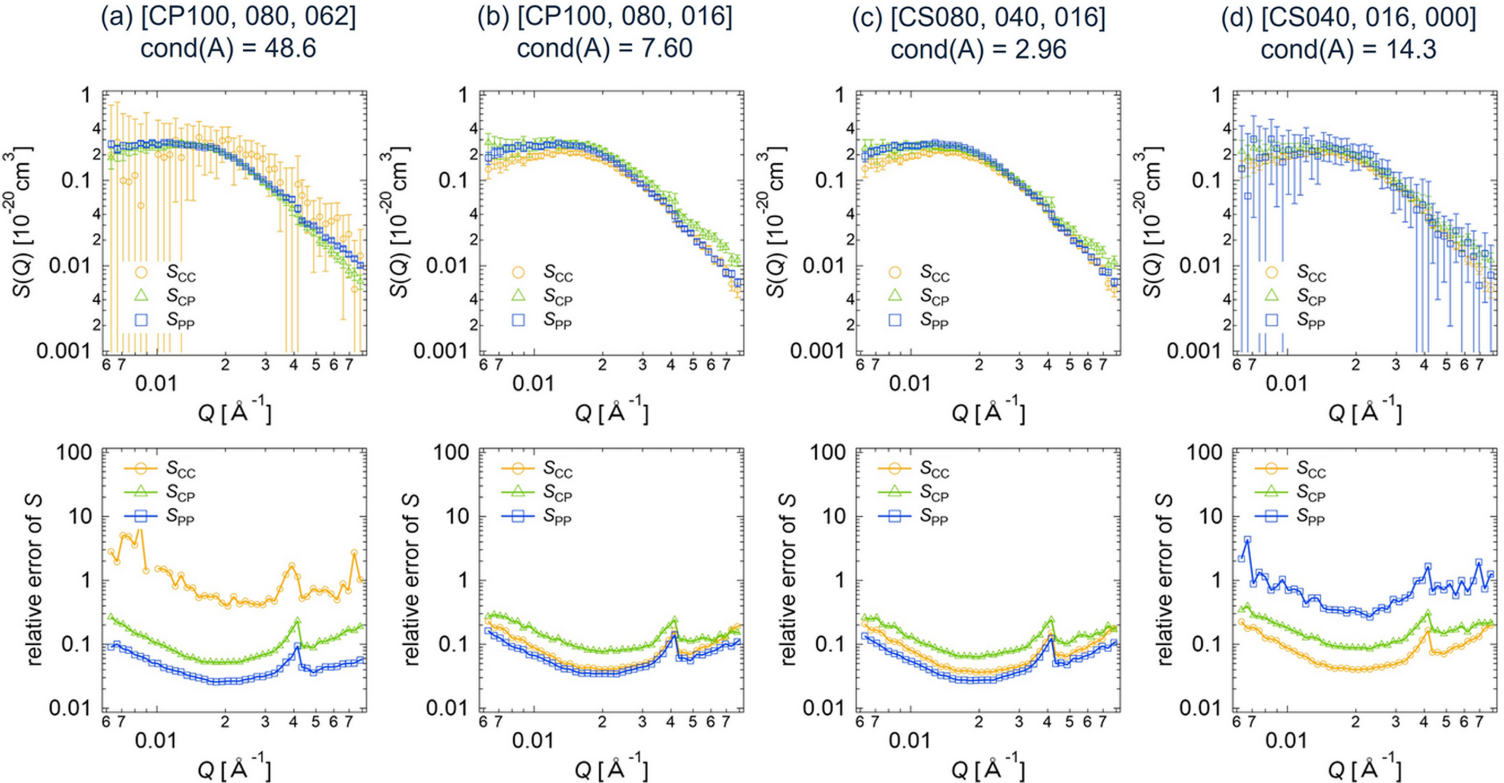
Partial scattering functions with error bars and their relative errors for the clay/PEG solutions, obtained by applying the deterministic error estimation method to the different data sets of the SANS scattering intensities: (*a*) CP100, CP080, CP062; (*b*) CP100, CP080, CP016; (*c*) CP080, CP040, CP016; (*d*) CP040, CP016, CP000.

In the same way as the deterministic estimation for the core–shell sphere (Section 3.1.1), we calculated the partial scattering functions and their errors for the clay/PEG solutions from various combinations of three data sets among the six with different scattering contrasts. Figs. 9 ▸(*a*)–9 ▸(*d*) show the calculated partial scattering functions and their relative errors for different contrast combinations, corresponding to condition numbers of *A* from 2.96 to 48.6. The obtained partial scattering functions are almost the same for all cases, while the relative errors in the partial scattering functions vary depending on the contrast combination. The cross-term *S*_CP_(*Q*) is positive, indicating that the PEG chains are adsorbed onto the clay particles (Matsunaga *et al.*, 2010 ▸).

#### 3.2.2. Statistical error estimation for clay/PEG solutions

**Figure 10:**
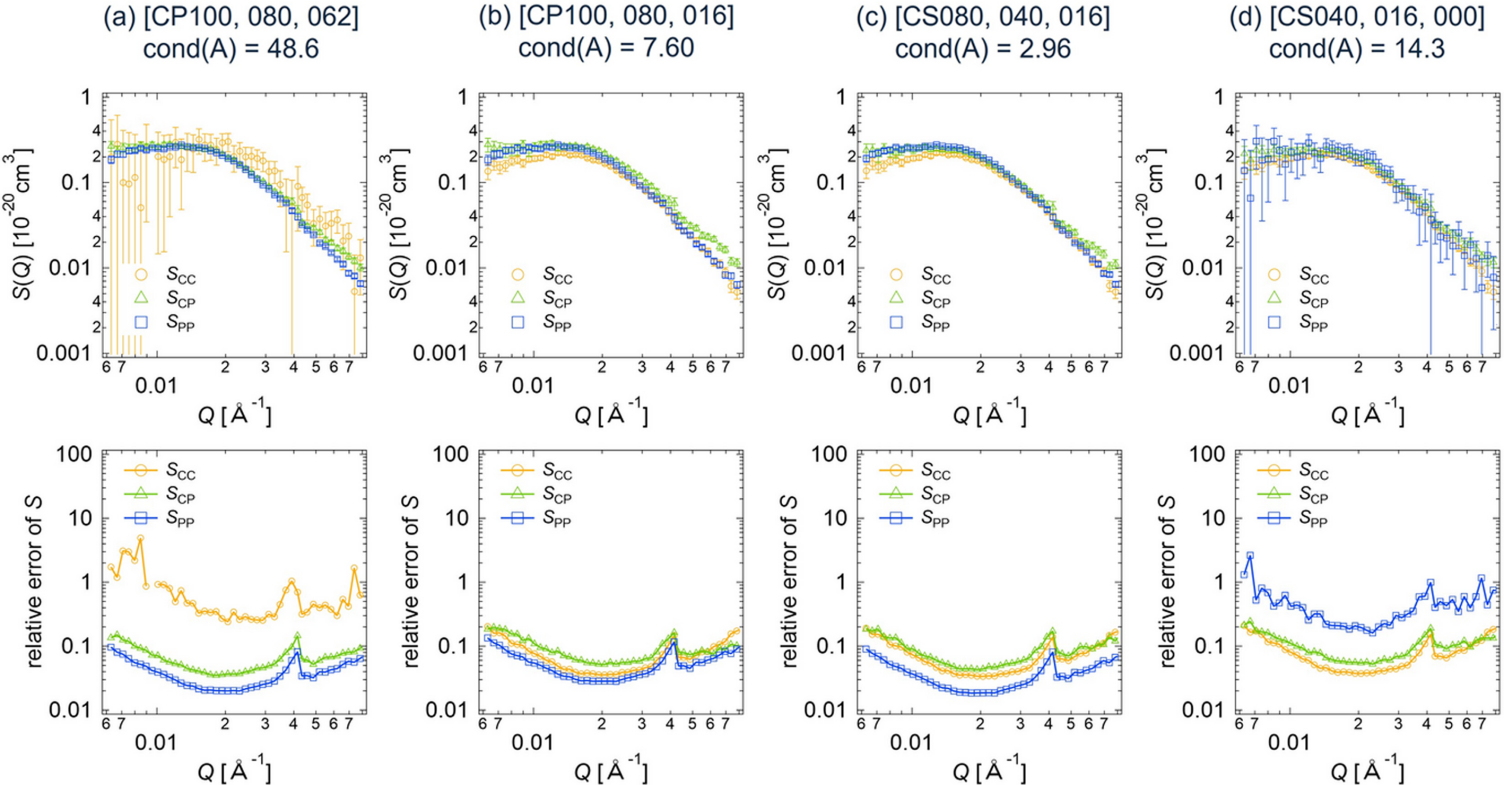
Partial scattering functions with error bars and their relative errors for the clay/PEG solutions, obtained by applying the statistical error estimation method to the different data sets of the SANS scattering intensities: (*a*) CP100, CP080, CP062; (*b*) CP100, CP080, CP016; (*c*) CP080, CP040, CP016; (*d*) CP040, CP016, CP000.

Here, we present the statistical error estimation results for the clay/PEG solutions. For this analysis, we set *I*_1_,…, *I*_*N*_ to the circularly averaged scattering intensities and σ_1_,…, σ_*N*_ to the standard deviations of these averages. Fig. 10 ▸ shows the partial scattering functions and their relative errors, computed using equation (11) for the different contrast combinations (*a*) to (*d*). The partial scattering functions obtained through the statistical method are quite similar to those obtained using the deterministic method, which are shown in Fig. 9 ▸.

#### 3.2.3. Comparison between deterministic and statistical error estimation results for clay/PEG solutions

**Figure 11:**
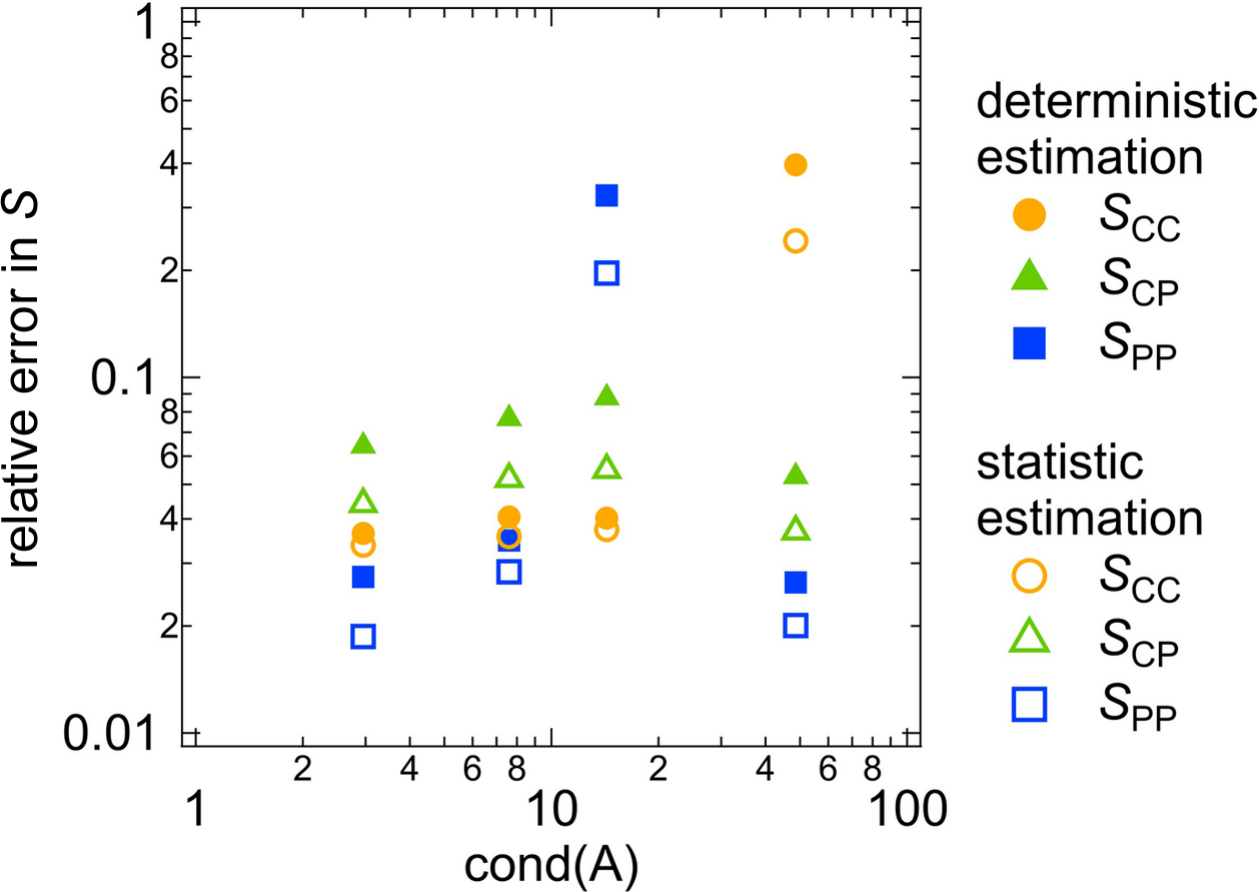
Plot of the relative errors in *S* versus the condition number of *A* for the clay/PEG solutions.

Fig. 11 ▸ displays the relationship between the condition number of *A* and the relative errors in the partial scattering functions *S* for the clay/PEG solutions at *Q* = 0.02 Å^−1^. This is similar to what was observed for the core–shell sphere (Fig. 7 ▸). The relative errors calculated by the statistical method are smaller than those obtained with the deterministic estimation method. Additionally, reducing the condition number of *A* decreases the relative errors in *S*. When the condition number of *A* is 2.96 or 7.60, the relative errors in *S* are less than 0.1 and all three partial scattering functions are determined with high accuracy. In contrast, for cond(*A*) = 14.3 or 48.6, the relative error in at least one partial scattering function exceeds 0.2, resulting in the large error bars in Figs. 9 ▸(*a*)–9 ▸(*d*) and Figs. 10 ▸(*a*)–10 ▸(*d*).

### 3.3. Error estimation for experimental data of PR solutions

**Figure 12:**
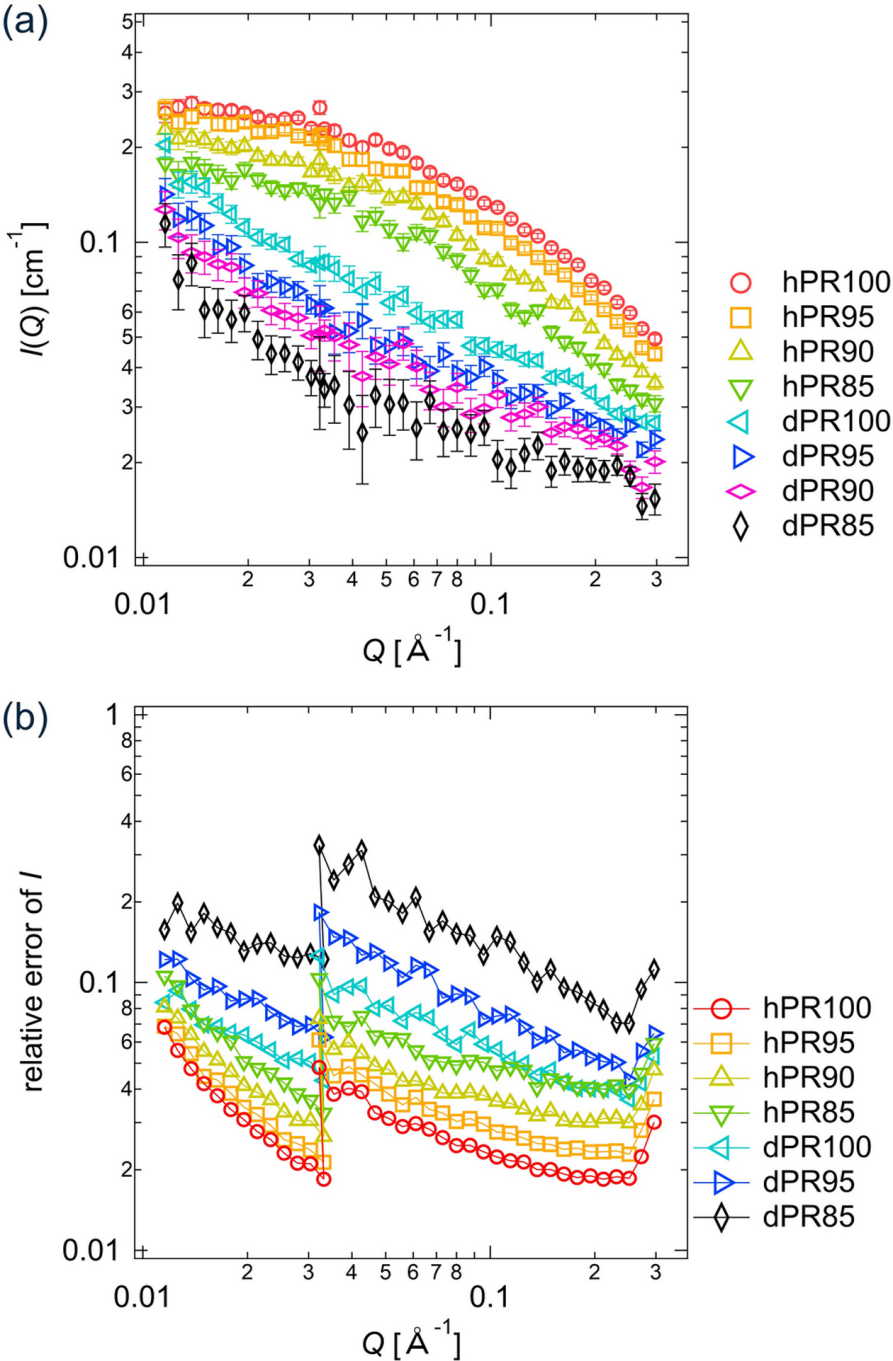
(*a*) Experimentally measured scattering intensities *I*(*Q*) with error bars and (*b*) relative errors in *I*(*Q*) for the PR solutions: hPR100, hPR095, hPR090, hPR085, dPR100, dPR095, dPR090 and dPR085.

Figs. 12 ▸(*a*) and 12 ▸(*b*) show the scattering intensities *I*(*Q*) and relative errors in *I*(*Q*), σ/*I*(*Q*), for the PR solutions with different scattering contrasts (Mayumi *et al.*, 2009 ▸). The scattering intensities *I*(*Q*) of the PR solutions are described by
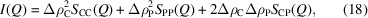
where *S*_CC_(*Q*) is the self-term for CD, *S*_PP_(*Q*) is the self-term for PEG and *S*_CP_(*Q*) is the cross-term between CD and PEG. For the PR solutions, *S*_11_, *S*_22_, *S*_12_, Δ_*i*_ρ_1_ and Δ_*i*_ρ_2_ for *i* = 1, …, *N* in equation (5) correspond to *S*_CC_, *S*_PP_, *S*_CP_, Δρ_C_ and Δρ_P_, respectively.

**Figure 13:**
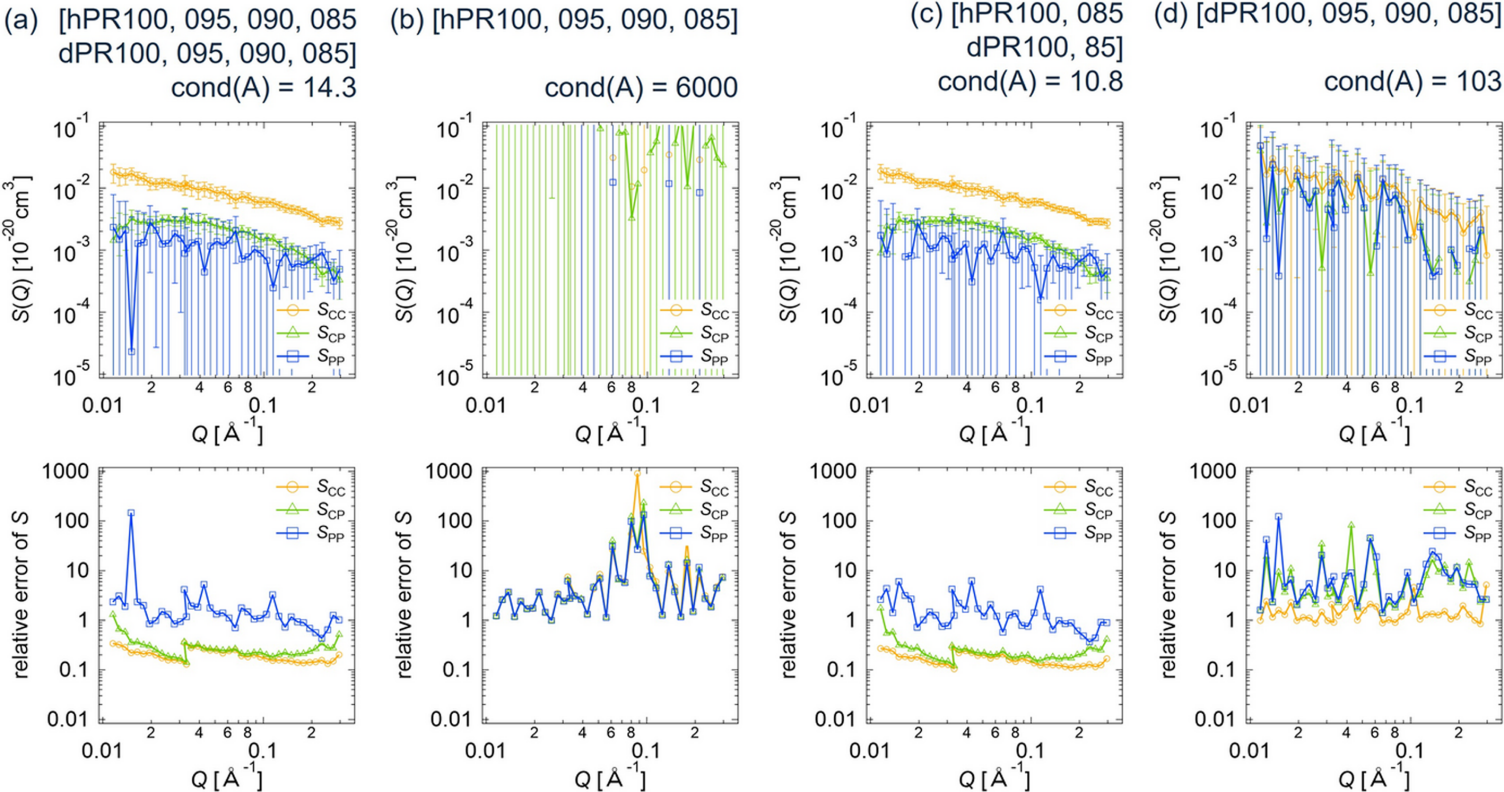
Partial scattering functions with error bars and their relative errors for the PR solutions, obtained by applying the deterministic error estimation method to the different data sets of the SANS scattering intensities: (*a*) hPR100, hPR095, hPR090, hPR085, dPR100, dPR095, dPR090, dPR085; (*b*) hPR100, hPR095, hPR090, hPR085; (*c*) hPR100, hPR085, dPR100, dPR085; (*d*) dPR100, dPR095, dPR090, dPR085.

In the same manner as for the CV-SANS data of the clay/PEG solutions, we performed the deterministic and statistical error estimations for the PR solutions. Figs. 13 ▸ and 14 ▸ show the partial scattering functions and their relative errors for the PR solutions using the deterministic and statistical methods, respectively. For cases (*a*) in Figs. 13 ▸ and 14 ▸, we used all eight SANS data sets (four contrasts of h-PR solutions and four contrasts of d-PR solutions). In this case, *S*_CC_(*Q*) and *S*_CP_(*Q*) are determined with sufficient accuracy. The positive cross-term *S*_CP_ represents the topological connection between CD and PEG (Mayumi *et al.*, 2009 ▸; Endo *et al.*, 2011 ▸). *S*_CC_, corresponding to the alignment of CD on PEG, can be described by a random copolymer model (Mayumi *et al.*, 2009 ▸; Endo *et al.*, 2011 ▸). However, the relative error in *S*_PP_(*Q*) is greater than 1, making it difficult to discuss the structure of PEG in PR on the basis of *S*_PP_(*Q*).

For cases (*b*) and (*d*), where only four data sets of h-PR or d-PR were used, the relative errors in all partial scattering functions exceed 1, indicating that both h-PR and d-PR data sets are necessary to reduce the error propagation. As shown in Figs. 13 ▸(*c*) and 14 ▸(*c*), when two sets of h-PR solution data (hPR100 and hPR085) and two sets of d-PR solution data (dPR100 and dPR085) were analyzed, the relative errors in the partial scattering functions were almost the same as those obtained from the eight-contrast case [Figs. 13 ▸(*a*) and 14 ▸(*a*)].

**Figure 15:**
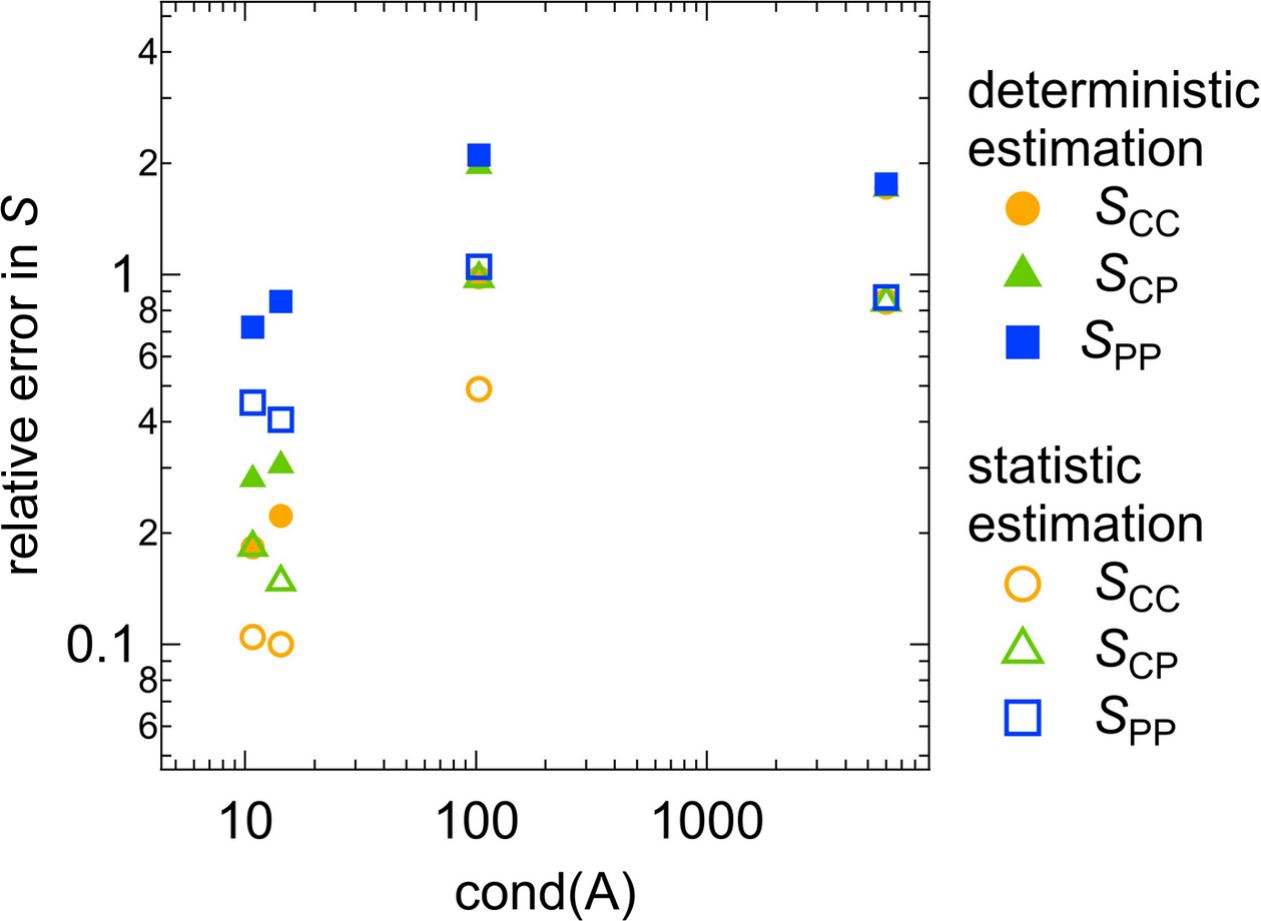
Plot of the relative errors in *S* versus the condition number of *A* for the PR solutions.

In Fig. 15 ▸, the relative errors in the partial scattering functions *S* of the PR solutions at *Q* = 0.02 Å^−1^ are plotted against the condition numbers of *A* for the different contrast combinations. In the same manner as for the core–shell sphere systems and clay/PEG solutions, minimizing the condition number of *A* is important for more precise determination of the partial scattering functions. Cases (*a*) and (*c*) in Figs. 13 ▸ and 14 ▸ correspond to small condition numbers of around 10, resulting in similar error estimation results for the two cases.

## 4. Conclusion

In this study, we have established deterministic and statistical error estimation methods for calculating partial scattering functions from scattering intensities of CV-SANS data. By applying these methods to (i) computational data of a core–shell sphere and experimental CV-SANS data of (ii) clay/polyethylene glycol aqueous solutions and (iii) polyrotaxane solutions, we have successfully achieved theoretically grounded error estimations of their partial scattering functions. This approach is valuable for evaluating the reliability of partial scattering functions computed from CV-SANS data. The statistical error estimation requires more assumptions than the deterministic error estimation, but the former usually gives sharper results than the latter. Therefore, the statistical error estimation is better if the assumptions are valid; otherwise, the deterministic approach is better.

This study has also highlighted the significance of the singular values of the matrix *A* appearing on the right-hand side of problem (4) in predicting error bars of partial scattering functions. For both the deterministic and statistical methods, the inverse of the minimum singular value 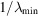 provides the scaling factor of absolute errors from CV-SANS measurements to the partial scattering functions, while the condition number 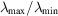 gives the scaling factor of relative errors. Because the condition number of *A* can be calculated only from scattering length densities ρ_*i*_, the scaling factor can be estimated before CV-SANS measurements. Therefore, by minimizing the condition number of *A*, we can optimize the choice of contrasts to reduce error propagation in the CV-SANS data analysis.

Additionally, these error estimation approaches can be used to reduce the number of samples required and shorten SANS measurement times. For example, Figs. 13 ▸ and 14 ▸ demonstrate that only four CV-SANS experimental data sets [case (*c*), cond(*A*) = 10.8] provide almost the same error bars for *S*_CC_(*Q*) and *S*_CP_(*Q*) as all eight CV-SANS experimental data sets [case (*a*), cond(*A*) = 14.3]. This fact suggests the possibility of reducing experimental costs using condition numbers.

## Figures and Tables

**Figure 14:**
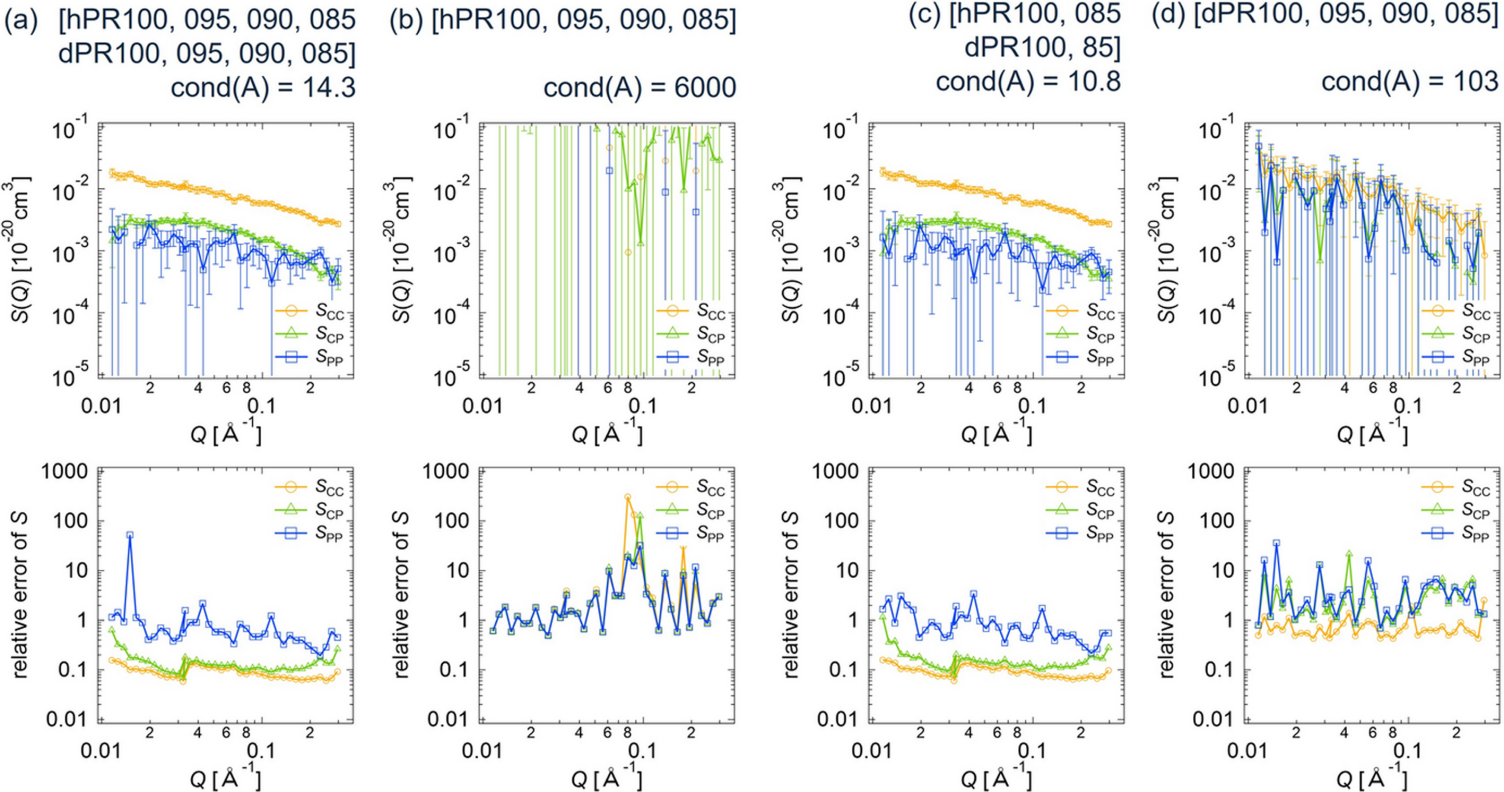
Partial scattering functions with error bars and their relative errors for the PR solutions, obtained by applying the statistical error estimation method to the different data sets of the SANS scattering intensities: (*a*) hPR100, hPR095, hPR090, hPR085, dPR100, dPR095, dPR090, dPR085; (*b*) hPR100, hPR095, hPR090, hPR085; (*c*) hPR100, hPR085, dPR100, dPR085; (*d*) dPR100, dPR095, dPR090, dPR085.

**Figure 16:**
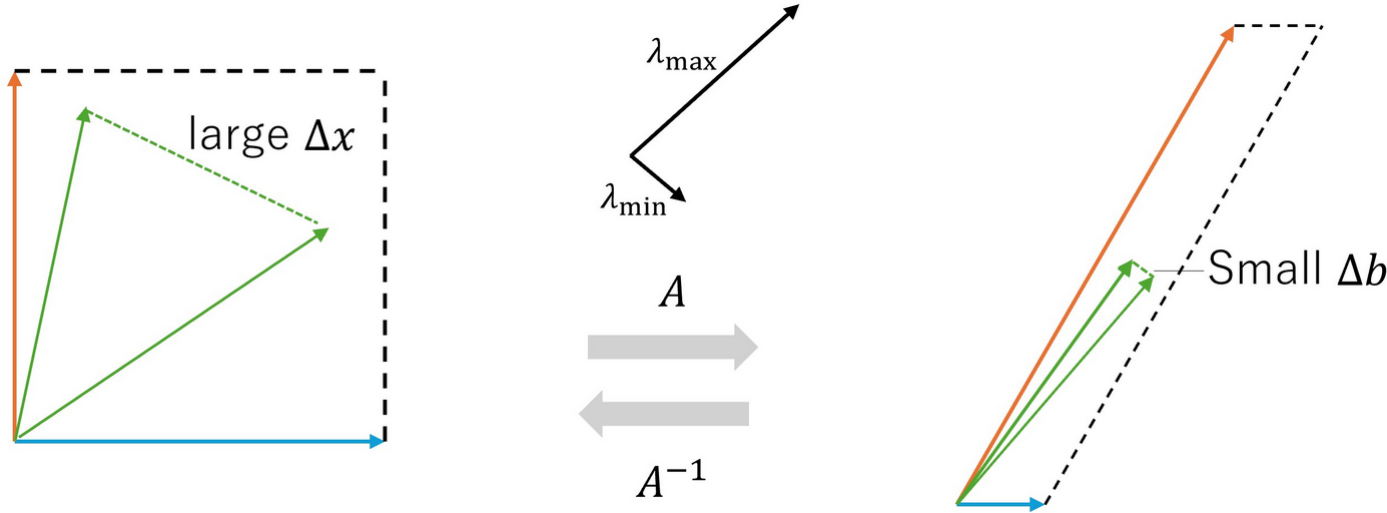
Illustration of how the matrix *A* transforms vectors.

## Appendix A Proofs of theorems

Proof of Theorem 1 The assumptions *AS* = *I* and *I* + Δ*I* = *A*(*S* + Δ*S*) imply *A*Δ*S* = Δ*I*. Since *A* has full column rank, we have Δ*S* = *A*^+^Δ*I*, so |Δ*I*| ≤ Δ*J* yields

Proof of Theorem 2 Since , we will estimate the upper bound of . Using the minimum singular value, we have the relation

Since *A*^T^Σ^−1^*A* is symmetric, the minimum singular value can be estimated as

where

The above provides the desired inequality. 

## Appendix B Condition numbers of matrices

The condition number of a matrix *A*, denoted cond(*A*), can be defined in two equivalent ways:

where *A*^+^ denotes the Moore–Penrose inverse of *A*, and  and  denote the maximum and minimum singular values of *A*, respectively. For symmetric positive definite matrices, singular values coincide with eigenvalues. The condition number of *A* is a measure of the sensitivity of the solution to a linear system involving the coefficient matrix to changes or errors in the input data. A high condition number indicates that the matrix is close to being singular or ill-conditioned, implying that small changes in the input can lead to large changes in the output.

To understand this intuitively, consider a system *Ax* = *b* with a 2 × 2 symmetric positive definite matrix *A* and how *A* transforms vectors. In Fig. 16 ▸, the square (0, 1)^2^ ∋ *x* on the left exists in the solution space and is transformed into the parallelogram *Ax* on the right in the image of *A*. Since *A* is symmetric positive definite, the singular values  and  are also eigenvalues. The eigenvalues  and  indicate maximum and minimum stretching, respectively. When *A* has a large condition number, a small change Δ*b* in the transformed space corresponds to a large change Δ*x* in the solution space.

For example, consider the symmetric positive definite matrix

This matrix transforms vectors by stretching them  = 3.9 times in one direction, while compressing them to  = 0.1 times in another direction. The ratio of these stretching factors defines the condition number cond(*A*) =  = 39. This large condition number implies that when solving *Ax* = *b* a small relative error of 1% in *b* can result in a much larger relative error of up to 39% in *x*.

This concept is generalized to higher dimensions, including the case of least-squares problems where *A* is not a square matrix. In such cases, instead of eigenvalues, we use singular values of *A* (which are the square roots of the eigenvalues of *A*^T^*A*) as key indicators. The following relationships help us understand how relative error propagation is governed by cond(*A*) in more general situations:

When considering a perturbation Δ*b* in *b* that leads to a change in the solution,

Combining these inequalities yields

Note that, when *A* is a square matrix with an ordinary inverse, *A*^+^ coincides with *A*^−1^. This inequality shows that, even when the relative error in *b* is small, it can be amplified by up to a factor of the condition number in the solution *x*. Although the geometric interpretation in Fig. 16 ▸ is not immediately obvious for nonsymmetric or rectangular matrices, this derivation shows why the condition number still governs the amplification of relative errors in the solution. For a detailed theoretical treatment, see Golub & Van Loan (2013 ▸).
